# A multicentre, open-label, observational local study to evaluate the low-density lipoprotein cholesterol-lowering effect of ezetimibe as prescribed in daily routine practice in the South African population

**Published:** 2007-07

**Authors:** Frederick Raal, Colin Schamroth, Jai Patel, Piet Becker

**Affiliations:** Department of Medicine, Division of Endocrinology and Metabolism, University of the Witwatersrand, Johannesburg; Milpark Hospital, Johannesburg; St Augustine's hospital, Durban; Medical Research Council, Pretoria

## Abstract

**Objective:**

The study examined the efficacy, safety and tolerability of co-administering the cholesterol absorption inhibitor, ezetimibe 10 mg with ongoing statin therapy in hypercholesterolaemic patients.

**Patients and methods:**

In this multicentre, open-label, observational study, hypercholesterolaemic patients (from 44 South African speciality practices) on statin therapy were screened and after meeting the inclusion criteria, all received ezetimibe (10 mg/day) in addition to their ongoing statin therapy for four weeks.

**Results:**

In 358 patients, ezetimibe co-administered with ongoing statin therapy significantly reduced the low-density lipoprotein cholesterol (LDL-C) level by an additional 21.9% in the total population. In the secondary-prevention patients (category 1 cardiovascular risk according to ESC guidelines adopted for South Africa), an additional 20.4% reduction was observed, and a 25.5% additional reduction for the primary-prevention patients (category 2 cardiovascular risk according to ESC guidelines adopted for South Africa). These results were consistent across gender, race, age, statin brand and dose subgroups. Ezetimibe co-administered with ongoing statin therapy also increased the number of patients reaching their LDL-C goals according to their risk category (2.5 mmol/l for category 1 patients and 3.0 mmol/l for category 2 patients). Ezetimibe-plus-statin therapy was well tolerated, with a good safety profile.

**Conclusion:**

Ezetimibe co-administered with ongoing statin therapy consistently produced significant additional improvements in LDL-C levels and goal attainment. This was observed for the whole population as well as for the two risk categories. The addition of ezetimibe to ongoing statin therapy may be considered for patients not achieving their LDL-C goals on conventional statin therapy.

## Summary

Circulating cholesterol, particularly low-density lipoprotein cholesterol (LDL-C), is a major risk factor for the development of coronary heart disease (CHD). Epidemiological studies have demonstrated a continuous and graded relationship between plasma total cholesterol (TC) and LDL-C concentrations and risk of death from CHD.^1^ Moreover, in both primary- and secondary-prevention trials, reductions in TC and LDL-C concentrations have been shown to result in substantial reductions in CHD morbidity and mortality.^2^-^9^ In view of the relationship between plasma LDL-C concentrations and CHD and the well-documented benefits of reducing LDL-C, current guidelines emphasise LDL-C lowering as a primary risk-reduction strategy.^10^-^12^

Although several classes of pharmaceutical agents have been approved for use as cholesterol-lowering agents, HMG-CoA reductase inhibitors or ‘statins’ are the most efficacious and are currently regarded as standard, first-line therapy worldwide for the treatment of hypercholesterolaemia. At their maximum doses, statins alone produce mean LDL-C reductions in the range of 50 to 60%, and modest reductions in triglycerides (TG) and elevations of high-density lipoprotein cholesterol (HDL-C) concentrations. HMG-CoA reductase inhibitors or statins have been marketed since 1988 and over 20 million patients have been treated with these agents at doses of 5 to 80 mg.

Despite the LDL-C-lowering efficacy of statins, a significant portion of the statin-treated hypercholesterolaemic population fails to reach therapeutic goals for LDL-C concentrations as defined by the ESC guidelines adopted for South Africa.^13^,^14^ This is attributed to the extent of cholesterol lowering required in some individuals as well as the lack of use of adequate doses of statins due to a variety of factors, including drug interactions, safety and tolerability issues. Statins also have limitations with regard to efficacy of LDL-C lowering, each doubling of the statin dose only achieving on average a further 6% LDL-C-lowering effect, commonly referred to as the ‘rule of 6’. Moreover, statins have limited efficacy in terms of other lipid abnormalities, including elevated TG and reduced HDL-C concentrations that often accompany hypercholesterolaemia and are believed to also affect CHD risk.

The European guidelines update the South African lipid guidelines published in February 2000.^15^ With limited healthcare resources, therapy has to be prioritised to those at highest risk, a concept which the European guidelines support. The LDL cholesterol goals of therapy are lower in those with established cardiovascular disease and those at higher risk.^14^

● Patients with established cardiovascular disease, type 2 diabetes or type 1 diabetes with microalbuminuria or those with severe genetic lipid disorders, such as familial hypercholesterolaemia, are at high risk and require intensive lifestyle intervention and drug therapy. The treatment goals for these patients are a total cholesterol of below 4.5 mmol/l and an LDL cholesterol of below 2.5 mmol/l (these patients are referred to as category 1).
● In asymptomatic, apparently healthy subjects, the decision to institute drug treatment depends not only on the lipid levels but, more importantly, on the assessment of cardiovascular risk. The treatment goals are a total cholesterol of below 5 mmol/l and LDL cholesterol of below 3 mmol/l. However, in these patients, if CVD risk remains high despite lifestyle intervention and initial drug therapy, one should consider therapy to lower total cholesterol to below 4.5 mmol/l and LDL cholesterol to below 2.5 mmol/l (these patients are referred to as category 2 patients).

In view of the limited efficacy of currently available treatments, new agents or combinations of agents with complementary effects are currently being sought to bridge the gap between LDL-C and TC treatment goals and outcomes. Ezetimibe is the first member of a new class of cholesterol-lowering agents that acts through inhibition of intestinal cholesterol absorption. Ezetimibe has been developed for use either as monotherapy or in combination with statins for LDL-C lowering in patients with primary, familial and non-familial hypercholesterolaemia.^16^

Ezetimibe inhibits the intestinal absorption of cholesterol and structurally related phytosterols by blocking their passage across the intestinal wall. Ezetimibe blocks a highly specific cholesterol transporter, identified as the Nieman Pick C1L1-like protein,^17^ in the intestine, resulting in decreased absorption of dietary and biliary cholesterol via the exogenous pathway without effects on the absorption of other lipids (eg, triglycerides), lipid derivatives (eg, bile acids), or lipid-soluble nutrients or vitamins.

Upon ingestion, ezetimibe is rapidly absorbed and undergoes quick and extensive glucuronidation in the small intestine and liver. Ezetimibe and its glucuronide conjugate, both of which are active as cholesterol absorption inhibitors, are routed to the liver via the enterohepatic circulation and returned to the small intestine, their site of action, via the bile. There is relatively little release of ezetimibe into the peripheral circulation. Within the circulation, ezetimibe is largely (> 98%) bound to plasma proteins. Therefore there is little distribution of the drug in peripheral tissues not involved in its excretion. The major route of excretion is the faeces, with little excretion in the urine. In radio-labelled drug tracer studies in humans, 78% was recovered in the faeces, with the balance secreted in the urine.^16^

The pharmacokinetic properties of ezetimibe have been investigated in multiple studies. The most effective dose is 10 mg daily. Higher doses do not lower LDL-C significantly further and the marketed dose is therefore 10 mg daily.

Importantly, ezetimibe is not an inhibitor or inducer of cytochrome P-450 (CYP450), reducing the potential for drug−drug interactions. This attribute renders ezetimibe a particularly appealing candidate for concomitant administration with statins, which are substrates for CYP450 enzymes and, when used concomitantly with drugs that are potent inhibitors of these enzymes, result in higher blood levels and increased risk of myopathy.^16^

## Patients

From the cardiology practices in the four main metropolitan areas, Johannesburg, Pretoria, Cape Town and Durban, 44 patients were prepared to participate in the study and, in anticipation of drop-outs, it was decided to include all of them, as the calculated sample size required the inclusion of 42 practices. Consenting patients who met all eligibility criteria and completed the screening phase successfully received ezetimibe 10 mg in addition to their ongoing statin therapy.

Male and female patients, aged 18 to 80 years, with hypercholesterolaemia who had been receiving a stable dose of statin therapy for a minimum of six weeks were eligible for enrolment into the study. Patients were excluded from the study if they had unstable angina or had suffered a myocardial infarction or undergone coronary artery bypass surgery or coronary angioplasty within the preceding three months. Other major exclusion criteria included a history of hypersensitivity to statin therapy, current active liver disease [liver transaminases (ALT or AST) > 1.5 times the upper limit of normal (ULN), or serum creatine kinase > 1.5 3 ULN]. Patients with impaired renal function, type 1 or 2 diabetes mellitus or uncontrolled hypothyroidism were also excluded.

## Study design

The study was a multicentre, open-label study to evaluate the LDL-C-lowering effect of ezetimibe when co-administered with ongoing statin therapy. After informed consent was obtained, demographic data were collected, cardiovascular risk factors were assessed and a fasting lipid profile and biochemistry levels were determined. Successfully enrolled patients returned for a dispensing visit to receive ezetimibe 10 mg once daily for four weeks.

The dose of the statin remained unchanged as per the patient’s previous therapy regimen. Patients were instructed not to change their dietary regimens or lifestyle in any manner during the course of the study. Each subject received a minimum of four weeks of active treatment. Fasting lipid profiles and biochemistry tests were repeated following the four weeks of therapy and any adverse events were recorded.

Patients were also contacted via telephone two weeks after the final visit to follow up on any adverse experiences. Participating physicians were paid the standard consultation fee for each visit and the sponsor paid for the laboratory tests. Patients were paid travel and meal costs according to MCC guidelines. Full ethics approval was obtained for the study and all patients were required to sign a consent form.

To avoid laboratory error, all blood tests were done centrally in a single laboratory.

## Statistical considerations

To determine the sample size, the conservative route, ie, maximum sampling was followed to assume a response rate of 50%, where response meant a lowering of at least 16% in LDL-C. A sample of 385 patients was required to determine the response rate to an accuracy of 5% (nQuery Advisor 6.0). To compensate for possible clustering within practices, a small intra-class correlation of 0.01 was assumed so that the design effect was 1.09 for a proposed cluster size of 10 patients, and 42 practices needed to be included in the study.

Demographic variables, cardiovascular risk factors and any changes in lipid levels (baseline and post TC, HDL-C, LDL-C and TG) were summarised using descriptive statistics, namely, mean, standard deviation, range and 95% confidence intervals. The success rate was calculated along with a 95% confidence interval and testing was done using the one-sample *t*-test at the 0.05 level of significance. Clustering of patients in practices was compensated for by taking into account the design effect.

## Results

A total of 501 patients were assessed for eligibility and 143 were excluded. Most exclusions occurred because patients did not meet the inclusion criteria. The most common exclusion criteria were the presence of diabetes mellitus, elevations of the liver transaminases (ALT, AST) ≥ 1.5 3 ULN with no active liver disease, and CPK ≥ 1.5 3 ULN.

The remaining 358 patients were included in the study and received ezetimibe 10 mg in addition to their ongoing statin therapy. Demographics and baseline characteristics for the eligible patients are shown in Table 1. Using the South African guidelines for cardiovascular risk,^14^ the 358 eligible patients were grouped into category 1 patients to be treated to an LDL-C goal of ≤ 2.5 mmol/l and category 2 patients to be treated to an LDL-C goal of < 3.0 mmol/l.

**Table 1:** Patient Data And Demographics Including Subgroups And Risk Categories 1 And 2

*Paremeter*	*Eligible patients (n 5 358)*		*Risk category (n 5 253)*		*Risk category 2 (n 5 105)*	
Age (*n*, %)						
< 65 yrs	269	75.1%	175	69.2%	93	88.6%
> 65 yrs	89	24.9%	78	30.8%	12	11.4%
Gender (*n*, %)						
Female	110	30.7%	57	22.5%	54	51.4%
Male	248	69.3%	196	77.5%	51	48.5%
Body weight (mean, range)	82.7 kg	44–170 kg	84.7 kg	48–170 kg	78 kg	44–142 kg
Smoking (*n*, %)						
Yes	61	16.8%	42	16.6%	78	17.2%
No	297	83.0%	211	83.4%	87	82.8%
Documented heart disease (*n*, %)						
Yes (category 1)	253	70.7%				
No (category 2)	105	29.3%				
LDL-C (mean, range)	3.77 mmol/l	1.42–8.18	3.64 mmol/l	1.42–8.18	4.1 mmol/l	1.9–7.9
HDL-C (mean, range)	1.3 mmol/l	0.71–2.49	1.35 mmol/l	0.71–2.49	1.5 mmol/l	0.9–2.45
TG (mean, range)	1.84 mmol/l	0.43–16.18	1.84 mmol/l	0.43–16.2	1.88 mmol/l	0.46–6.63

Of the statin brands used at baseline (Table 2), atorvastatin was the most commonly used in both risk categories (66.8 and 65.7%), followed by simvastatin (20.9 and 17.1%). Daily statin doses ranged from 10 to 80 mg.

**Table 2:** Statin Brand Distribution

*Statin*	*Risk category 1 (n 5 253)*		*Risk category 2 (n 5 105)*	
	*n*	*%*	*n*	*%*
Atorvastatin	169	66.8	69	65.7
10 mg	13	7.7	23	33.3
20 mg	53	31.4	26	37.7
40 mg	79	46.7	16	23.2
80 mg	16	9.5	4	5.8
Other	8	4.7	0	0.0
Simvastatin	53	20.9	18	17.1
10 mg	11	20.8	3	16.7
20 mg	17	32.1	8	44.4
40 mg	22	41.5	6	33.3
80 mg	3	5.7	1	5.6
Other	0	0.0	0	0.0
Pravastatin	19	7.5	5	4.8
10 mg	2	10.5	0	0
20 mg	4	21.1	2	40
40 mg	12	63.2	3	60
80 mg	1	5.3		
Other	0	0.0		
Other statins	12	4.7	13	12.4
	253		105	

## Effectiveness results for LDL-C and additional parameters

Ezetimibe co-administered with ongoing statin therapy significantly reduced mean LDL-C levels by an additional 21.9% (95% CI: 19.3−24.4%), from 3.77 mmol/l to 2.90 mmol/l for the total population. The reduction in LDL-C for the patients at higher risk (risk category 1 according to the ESC guidelines) was an additional 20.4% when ezetimibe was added to the existing statin therapy. In the primary-prevention patients (risk category 2 according to the ESC guidelines) the additional mean reduction in LDL-C was 25.5%.

**Fig. 1.**
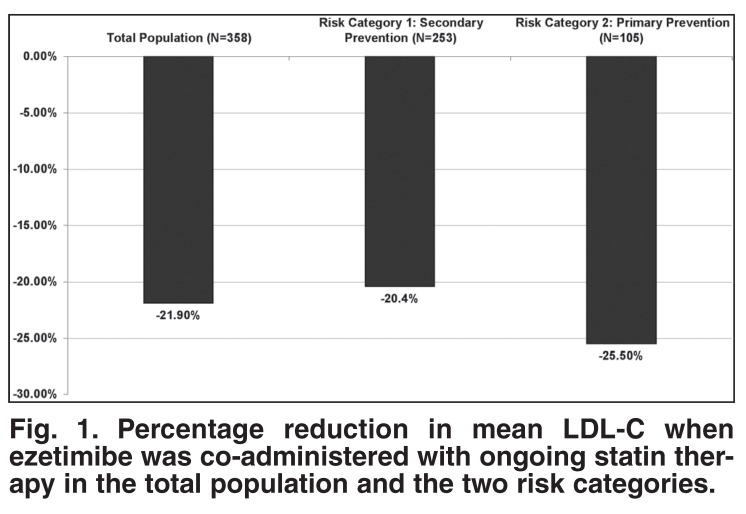
Percentage reduction in mean LDL-C when ezetimibe was co-administered with ongoing statin therapy in the total population and the two risk categories.

The percentage LDL-C reduction was significantly greater than 16% (*p* < 0.001), ie, criteria for treatment success. The response rate was 69.6% with 95% CI: 63.8−75.3%, which extends within 6% of the observed response rate. This result was maintained if the criterion of greater than 16% was changed to greater than 19% (*p* = 0.027). The mean percentage reduction in total cholesterol for the total study population was 18.1% (95% CI: 16.5−19.6%). There was little change in HDL cholesterol and triglycerides (Fig. 1).

**Fig. 2.**
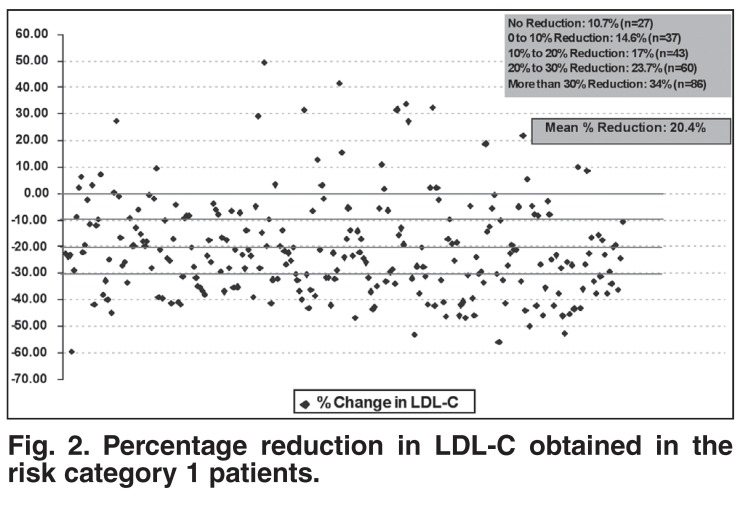
Percentage reduction in LDL-C obtained in the risk category 1 patients.

**Fig. 3.**
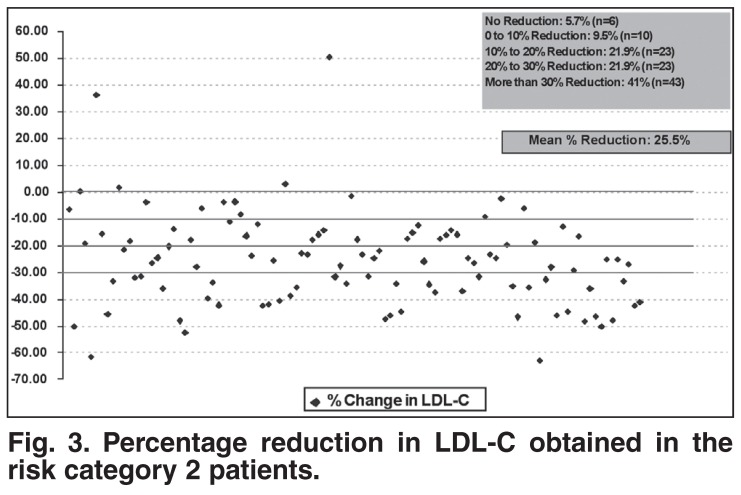
Percentage reduction in LDL-C obtained in the risk category 2 patients.

The reduction in LDL-C was consistent across risk categories and statin brands. In particular, for risk category 1 patients on atorvastatin (*n* = 160) and simvastatin (*n* = 52), it was 22.7 and 18.4%, while for risk category 2 patients on atorvastatin (*n* = 71) and simvastatin (*n* = 18), it was 26.7 and 24.1% (Table 3). In addition, although the mean LDL-C lowering for category 1 patients was 20.4%, more than 34% of patients achieved a greater than 30% further LDL-C reduction (Fig. 2). Similarly, in the risk category 2 patients, although the mean LDL-C reduction was 25.5%, more than 41% of patients achieved a greater than 30% further LDL-C reduction (Fig. 3).

**Table 3:** Percentage Reduction In LDL-C According To Statin Brand

	*Mean change (%)*	*95% CI (%)*
Population (*n* = 358)	21.9	19.3–24.4
Risk category 1 (*n* = 253)	20.4	17.5–23.4
Atorvastatin (*n* = 160)	22.7	19.8–25.6
Simvastatin (*n* = 52)	18.4	11.9–24.8
Risk category 2 (*n* = 105)	25.5	21.8–29.1
Atrovastatin (*n* = 71)	26.7	21.6–31.8
Simvastatin (*n* = 18)	24.1	16.1–32.1

**Fig. 4.**
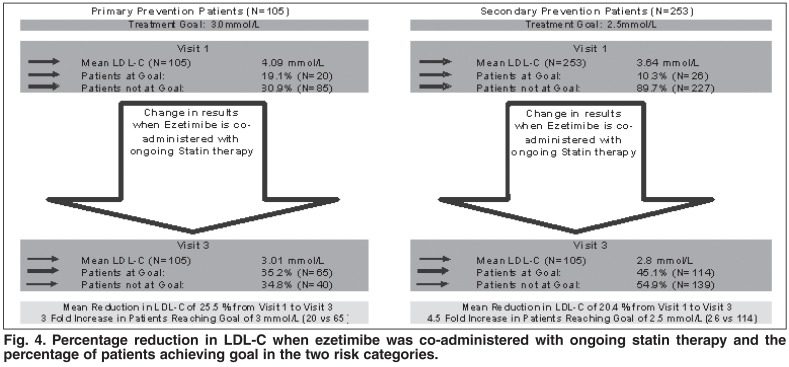
Percentage reduction in LDL-C when ezetimibe was co-administered with ongoing statin therapy and the percentage of patients achieving goal in the two risk categories.

Ezetimibe co-administered with ongoing statin therapy was effective in bringing 45.1% (114/253) of secondary-prevention patients (risk category 1) to an LDL cholesterol goal of 2.5 mmol/l, compared to the 10.3% (*n* = 26) of patients who were at goal on their statin therapy alone. Of the primary-prevention patients (risk category 2) with an LDL cholesterol treatment goal of 3.0 mmol/l, the patients at goal increased from 19.1% (*n* = 20) to 65.2% (*n* = 65) when ezetimibe was co-administered with ongoing statin therapy (Fig. 4).

The drop-out rate due to adverse events was very low (0.56%) and no serious adverse events were reported throughout the study.

## Conclusion

Ezetimibe 10 mg/day, when co-administered with ongoing statin therapy in patients who had not yet attained their LDL-C goals produced significant additional improvements in LDL-C levels, resulting in clinically significant increases in the proportion of patients who attained their LDL-C goals. These improvements in LDL-C levels were consistently observed across age and gender, and with all statin brands and doses studied in this protocol.

For patients not achieving their ESC lipid goals while receiving ongoing statin therapy, the addition of ezetimibe may be considered a safe and effective way to help such patients reach their LDL-C goal.
